# Nominal and achieved stromal ablation depth after myopic transepithelial photorefractive keratectomy: implications for residual stromal thickness calculation

**DOI:** 10.1186/s40662-024-00404-2

**Published:** 2024-09-02

**Authors:** Yue Feng, Tore Arnstein Nitter, Xu Liu, Aleksandar Stojanovic

**Affiliations:** 1 Institute of Community Medicine, Faculty of Health Sciences, University in Tromsø, Tromsø, Norway; 2 Department of Ophthalmology, University Hospital North Norway, Sykehusveien 38, 9019 Tromsø, Norway; 3 Institute of Clinical Medicine, Faculty of Health Sciences, University in Tromsø, Tromsø, Norway; 4 University Hospital of Northern Norway, Tromsø, Norway

**Keywords:** Transepithelial photorefractive keratectomy, Stromal ablation depth, Residual stromal thickness, Epithelial remodeling

## Abstract

**Background:**

The primary objective of this investigation was to compare the nominal central ablation depth with the achieved central corneal stromal ablation depth after StreamLight transepithelial photorefractive keratectomy (tPRK) for myopia with WaveLight® laser by Alcon Laboratories, TX, USA.

**Methods:**

This ambispective study encompassed a retrospective analysis of 40 eyes who underwent treatment for myopia and astigmatism, followed by a prospective examination conducted 6–9 months postoperatively. Pre- and postoperative Avanti spectral-domain optical coherence tomography (SD-OCT; Optovue Inc., CA, USA) provided stromal and epithelial thickness maps. The difference between pre- and postoperative central stromal thicknesses at the corneal vertex was used to calculate the achieved stromal thickness ablation depth. This value was then compared with the corresponding central nominal depth on the laser ablation planning map.

**Results:**

A total of 40 eyes (OD/OS:18/22) of 40 patients (31.4 ± 9.2 years) were available for evaluation. The mean treated spherical equivalent was − 2.98 ± 1.46 D. The mean nominal and achieved central stromal ablation depths were 51.22 µm and 59.67 μm, respectively, showing a mean stromal excessive ablation of 16.50%. The mean pre- and postoperative central epithelial thicknesses were 53.74 μm and 59.31 μm, respectively, showing a mean postoperative thickness increase of 10.46%. This increase in the epithelial thickness rendered the mean postoperative pachymetry reduction to 54.11 μm, only 2.33% greater than the mean nominal ablation depth.

**Conclusions:**

The study revealed a central stromal ablation 16.50% greater than the nominal ablation depth. This excessive stromal removal was largely compensated for by the increase in epithelial thickness, resulting in a mean difference between the nominal ablation depth and the achieved central corneal pachymetry reduction of only 2.33%. This significant excessive central stromal ablation must be taken into consideration in the calculation of the residual stromal thickness.

## Background

Understanding the relationship between achieved and nominal central stromal ablation as well as the increase in corneal epithelial thickness following myopic surgery is crucial for comprehending the induced structural corneal changes. The nominal depth is typically assumed to represent the depth of stromal ablation and is used in calculating the residual stromal thickness (RST). A disparity between these two measures may be particularly critical in instances of low preoperative corneal pachymetry where knowledge of RST is vital for ensuring surgical safety [1]. Excessive stromal ablation may elevate the risk of iatrogenic corneal ectasia development [2].

Several laser-assisted in situ keratomileusis (LASIK) studies have delved into the discrepancy between nominal and achieved laser ablation [3–6], primarily utilizing Scheimpflug imaging technology and /or time-domain optical coherence tomography (OCT) to compare total pre- and postoperative corneal pachymetry. However, the technology used in these studies did not allow the measurement of stromal thickness, which is crucial for estimating the reduction in corneal biomechanical strength.

The current study addresses the disparity between nominal and achieved stromal ablation depth in StreamLight (Alcon Laboratories, TX, USA) tPRK. Given the comparatively lower impact of photorefractive keratectomy (PRK) on corneal biomechanical stability compared with LASIK [7, 8], tPRK is presumably used more frequently than LASIK in cases with thin corneas. Therefore, identifying the correct amount of stromal tissue removal needed to correct the preoperative refractive error with tPRK is especially critical. The current study also compares and analyzes preoperative and postoperative central epithelial thicknesses, unraveling the effect of postoperative epithelial remodeling after tPRK.

## Methods

### Study design

This study employed an ambispective design, entailing a retrospective analysis of 40 cases treated for myopia and astigmatism by StreamLight tPRK, followed by a prospectively designed follow-up examination conducted 6–9 months postoperatively.

### Patients

Forty eyes from 40 subjects (randomly chosen between both eyes) who underwent tPRK for myopia at the Memira Eye Center in Tromsø, Norway, using the StreamLight treatment, were included in this study. Inclusion criteria were: 1. Age ≥ 18 years; 2. No soft contact lens wear for one week (four weeks for hard contact lenses) prior to the baseline examination; 3. Manifest spherical equivalent between –1.0 and –8.0 diopters (D) with ≤ 3.00 D of manifest astigmatism; 4. Stable refractive error, with a change in spherical equivalent refraction of ≤ 0.50 D during the last two years before surgery; 5. Corrected visual acuity better than 0.00 logMAR (logarithm of the minimum angle resolution) units. Exclusion criteria were: 1. Corneal pathology, including keratoconus or keratoconus suspect (detected by corneal topo/tomography); 2. Irregular astigmatism; 3. Moderate to severe dry eye; 4. Any posterior segment pathology or previous intraocular or corneal surgery; 5. Diabetes; 6. Systemic diseases that could affect corneal wound healing (e.g., collagen vascular diseases).

### Preoperative examination

Non-cycloplegic subjective and objective refraction assessments as well as evaluations of visual acuity, uncorrected and corrected distance visual acuity (UDVA, CDVA) using the logMAR 4 m chart (Sussex Vision, Inc., Rustington, UK) were performed. A comprehensive ophthalmologic examination followed, including corneal topography/tomography using Topolyzer and Oculyzer (Alcon Laboratories, Fort Worth, TX, USA), and corneal OCT scanning, which included epithelial thickness mapping using Avanti (Optovue Inc, Freemont, CA, USA) spectral-domain OCT (SD-OCT). Three successive OCT measurements were taken, and the average was considered as a representative value.

The preoperative examination served to determine suitability for myopic laser vision correction (LVC) surgery and to decide the required spherocylindrical correction and the depth of epithelial laser ablation to be programmed into the laser ablation planning software.

### Single-step transepithelial PRK procedure

#### Surgical protocol

The target refraction was 0.00 D (emmetropia) for all eyes.

#### Before surgery

The patient was instructed to take Omega-3 capsules (1000 mg daily) and Vitamin C tablets (1000 mg daily) for two weeks prior to surgery. Bromfenac (Yellox, Bausch + Lomb, NJ, USA) was prescribed at a dosage of one drop twice daily, starting two days before surgery.

### The surgical procedure

1. Five minutes prior to surgery, dexamethasone/chloramphenicol (Spersadex med Kloramfenikol, Laboratoires Théa, France), or an alternative steroid/antibiotic combination eye drops was administered.
2. The first anesthetic drop (proparacaine 0.5%, alcaine, Alcon) was given during eyelid and surgical field preparation using povidone iodine (betadine 5% sterile ophthalmic prep solution, Alcon).
3. Eyelashes and meibomian gland orifices were covered using a transparent film dressing (Tegaderm, 3 M, USA).
4. The second drop of alcaine was applied just before the insertion of the eye speculum.
5. Registration: a. Alignment of both eyes' pupils on the common x-line of the projected red laser-cross was achieved by patient's head adjustment. b. Verification of Topolyzer-imported x–y offset to achieve vertex centration of the operated eye was performed by pressing the laser's right-hand pedal. c. Patient's fixation was verified by identifying the Purkinje images of the green fixation light.
6. Prior to initiating eye-tracked laser ablation, a cooling with semi-frozen balanced salt solution (BSS) sterile irrigating solution (15 mL bottle; Alcon Laboratories, Fort Worth, TX, USA) was given. The previously frozen plastic bottle was microwaved at 700 W for 15 s right before the surgery, with most of the ice melting (leaving some ice fragments). Cooling was achieved by dispensing 10–15 drops directly from the bottle for 5.0–7.5 s. Excess fluid was then removed using the first lint-free, pre-expanded Merocel sponge to absorb most of the fluid towards the palpebral edge. Finally, the second Merocel was gently wiped over the corneal surface to achieve a uniform semi-moist surface.
7. Epithelial ablation based on the central thickness measured by the Avanti SD-OCT [The StreamLight software requires programming of the epithelial thickness with predetermined choices (45 µm, 50 µm, 55 µm, 60 µm, and 65 µm), we rounded the ablation depth to the nearest higher number, e.g., an epithelial thickness of 51–55 µm was programmed to 55 µm, an epithelial thickness of 56–60 µm was programmed to 60 µm]. Then, using the WaveLight® EX 500 excimer laser, an interruption by a 10-s break initiated by an audio signal from the laser machine was followed by stromal ablation with an optical zone of 6.5 mm for all eyes.
8. Semi-frozen BSS drops were applied for the second time within 5.0–7.5 s.
9. 0.02% mitomycin C was not utilized in any of the study cases.
10. A single eye drop of the Dexamethasone/Chloramphenicol combination and a single eye drop of bromfenac were administered.
11. A bandage contact lens (BCL) (Air Optix Night & Day, Alcon) was placed after the procedure and retained for 3–5 days until complete corneal epithelial healing was achieved.

### After surgery

The patient was advised to continue taking Omega-3 capsules (1000 mg daily) and Vitamin C tablets (1000 mg daily) for two months post-surgery. Bromfenac was continued at one drop twice daily for two days following surgery. Dexamethasone/chloramphenicol was prescribed at a dosage of one drop four times daily during the first week, three times daily during the second week, twice daily during the third week, and once daily during the fourth week after surgery.

### Postoperative examination

A postoperative examination was conducted on 40 eyes from 40 patients who had undergone treatment between 6 and 9 months before. The examination comprised an ophthalmologic assessment, including non-cycloplegic subjective and objective refraction, and evaluation of visual acuity. Corneal stromal and epithelial thickness mapping were performed using Avanti SD-OCT, with three successive measurements and the average used as a representative value.

### Anterior-segment optical coherence tomography

Pre- and postoperative Avanti SD-OCT examinations yielded stromal thickness maps. The measured difference between pre- and postoperative stromal maps was used to calculate the achieved stromal ablation depth at the corneal vertex, where all the treatments were centered. These values were then compared with the corresponding nominal values found on the ablation maps of the Alcon/WaveLight® laser ablation planning software, and the differences were analyzed.

The difference between pre- and postoperative epithelial thickness at the vertex showed the contribution of epithelial remodeling to the net change in corneal pachymetry.

### Statistical analysis

The statistical analysis employed SPSS 25.0 software (IBM Corp, Armonk, NY). The data distribution normality was confirmed through the Kolmogorov–Smirnov test. Paired samples t-tests and Wilcoxon signed-ranks tests were used to compare pre- and postoperative values. The difference (in percentage) between the nominal ablation depth and the achieved stromal ablation depth for each eye was calculated and then averaged; the same procedure was done for the nominal ablation depth and the corneal pachymetry reduction. Bland–Altman plots were generated to visualize the differences between the nominal ablation depth and A) achieved stromal ablation and B) corneal pachymetry reduction. The Pearson correlation coefficient was calculated to assess linear relationships. Statistical significance was defined for a *P* value of less than 0.05.

Sample size calculation was performed using PASS (Power Analysis and Sample Size) 15.0.5 Statistical Software (UT, USA), which involved comparing the achieved central stromal ablation depth to the nominal ablation depth. The Pearson correlation test was used to calculate the correlation between these two variables. With a significance level of 0.05 and a power of 90%, a sample size of 37 was found to be sufficient for detecting a difference of –0.50 between the null hypothesis correlation of 0.00 and the alternative hypothesis correlation of 0.50. Forty eyes were recruited in this study.

### Informed consent process

The research adhered to the principles of the Declaration of Helsinki. Informed consent was obtained before the examination and conducted following approval from the regional ethics committee REK-Nord (No. 311856).

## Results

Forty eyes (OD/OS: 18/22) of 40 patients (25 female, 15 male) with a mean age of 31.4 ± 9.2 years (range: 18 to 51 years) and a mean postoperative follow-up time of 7.4 ± 1.0 months (range: 6.2 to 9.0 months) were included in the evaluation. Patient demographics and preoperative data are presented in Table 1.

**Table 1:** Patient demographics and preoperative data

Parameters	Value
No. of eyes	40
No. of patients	40
No. of OD/OS	18/22
Age (years), mean ± SD (range)	31.4 ± 9.2 (18 to 51)
Gender (female/male)	25/15
Follow-up time (months), mean ± SD (range)	7.4 ± 1.0 (6.2 to 9.0)
LogMAR UDVA (mean ± SD)	0.82 ± 0.6
LogMAR CDVA (mean ± SD)	− 0.08 ± 0.07
MRSE (D), mean ± SD (range)	− 2.98 ± 1.46 (− 6.00 to − 1.00)
Central pachymetry (μm), mean ± SD (range)	520.1 ± 33.8 (459 to 578)
Central epithelial thickness (μm), mean ± SD (range)	53.74 ± 2.54 (49 to 63)
Nominal ablation depth (μm), mean ± SD (range)	51.22 ± 23.12 (15.68 to 99.74)

*SD* = standard deviation; *LogMAR* = logarithm of the minimum angle resolution; UDVA = uncorrected distance visual acuity; *CDVA* = corrected distance visual acuity; *MSER* = manifest refraction spherical equivalent

There were no complications, or adverse reactions observed in the 40 eyes.

**Fig. 1:**
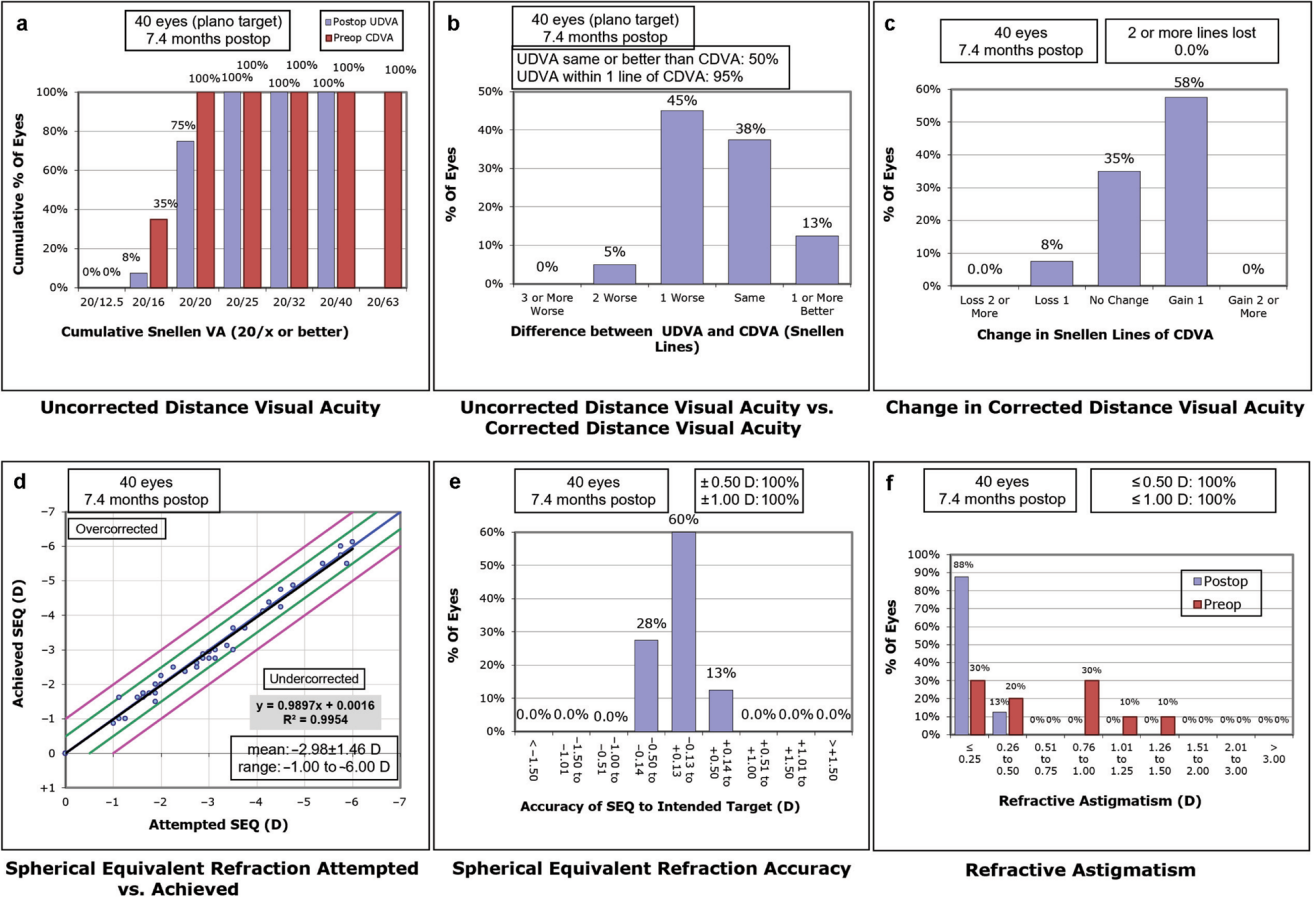
Six-standard graph for reporting refractive surgery showing the visual and refractive outcomes for 40 myopic eyes treated by the WaveLight® EX 500 excimer laser machine (Alcon Laboratories, TX, USA) using StreamLight® procedure. **a** Efficacy—postoperative uncorrected distance visual acuity (UDVA) versus preoperative corrected distance visual acuity (CDVA). **b** Difference between postoperative UDVA and preoperative CDVA. **c** Safety—change in CDVA preoperative versus postoperative. **d** Postoperative spherical equivalent refraction (SEQ) attempted versus achieved. **e** Accuracy of SEQ to intended target. **f** Refractive astigmatism postoperative versus preoperative. postop, postoperative; preop, preoperative; D, diopter

Figure 1 shows the refractive and visual outcome. The average efficacy and safety index were 0.88 ± 0.15 and 1.12 ± 0.16, respectively. The mean manifest refraction spherical equivalent postoperatively was − 0.04 D (− 0.50 D to + 0.50 D), with 100% within ± 0.5 D emmetropia. Table 2 summarizes the pre- and postoperative visual and refractive data.

**Table 2:** Pre- and postoperative visual and refractive data

Variables		Preoperative	Postoperative	*P* value
UDVA (logMAR)	Mean ± SD	0.82 ± 0.60	− 0.02 ± 0.10	*P* < 0.001
	Range	0.05 to 2.0	− 0.18 to 0.22	
CDVA (logMAR)	Mean ± SD	− 0.08 ± 0.06	− 0.13 ± 0.06	*P* < 0.001
	Range	− 0.18 to 0.00	− 0.18 to 0.00	
Spherical refraction (D)	Mean ± SD	− 2.51 ± 1.29	− 0.03 ± 0.29	*P* < 0.001
	Range	− 5.00 to − 1.00	− 0.50 to 0.50	
Cylindrical refraction (D)	Mean ± SD	− 0.96 ± 0.86	− 0.02 ± 0.35	*P* < 0.001
	Range	− 3.00 to 0.00	− 0.75 to 0.75	
MRSE (D)	Mean ± SD	− 2.98 ± 1.46	− 0.04 ± 0.21	*P* < 0.001
	Range	− 6.00 to − 1.00	− 0.50 to + 0.50	

*SD* = standard deviation; *logMAR* = logarithm of the minimum angle resolution; *UDVA* = uncorrected distance visual acuity; *CDVA* = corrected distance visual acuity; *MSER = *manifest refraction spherical equivalent

**Fig. 2:**
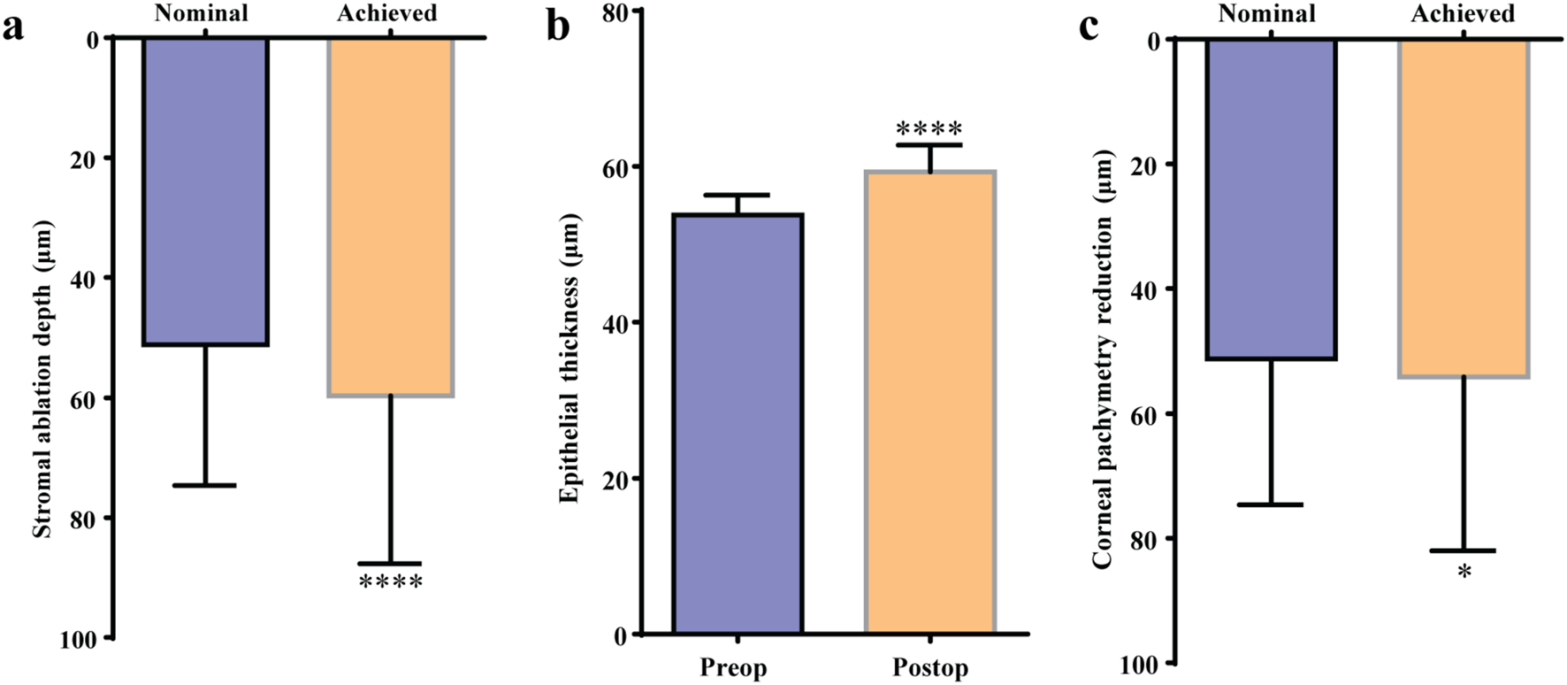
Bar graphics for corneal pachymetry and sublayers thickness. **a** The mean nominal ablation depth and achieved central stromal ablation depths (reduction). **b** The mean pre- and postoperative central epithelial thicknesses. **c** The mean nominal ablation depth and achieved corneal pachymetry reduction. ****, *P* < 0.001; *, *P* = 0.01

**Fig. 3:**
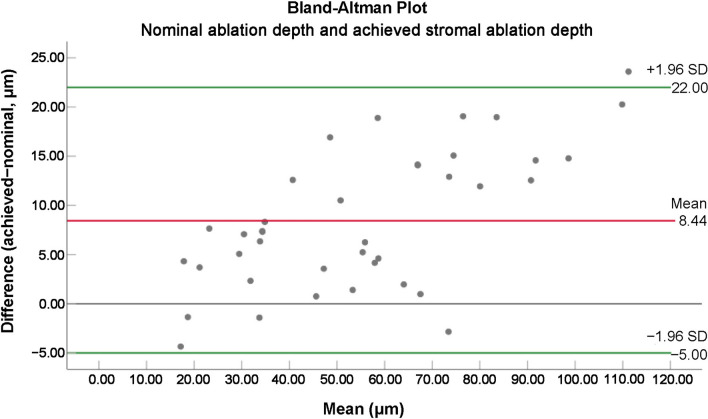
Bland–Altman plot for nominal ablation depth and achieved central stromal ablation depth. The red lines show the mean differences while the green lines show the lower and upper 95% limits of agreement

The mean central nominal (planned) ablation depth and achieved stromal ablation depths were 51.22 ± 23.12 μm and 59.67 ± 27.66 μm, respectively, signifying a mean achieved stromal ablation excess of 8.44 μm (*P* < 0.001) or 16.50% greater ablated in the central stroma vs. nominal. Nominal ablation depth and achieved corneal thicknesses (pachymetry and stroma) are presented in Table 3. Figure 2a illustrates the mean nominal ablation depth and achieved stromal ablation depth. Figure 3 shows the Bland–Altman plot for nominal ablation depth and achieved stromal thickness depth.

**Table 3:** Nominal ablation depth and achieved corneal thicknesses

Variables		Nominal	Achieved	Tissue gain/loss (+ / −)	Tissue gain/loss (+ / −) %	*P* value
Stromal ablation depth (μm)	Mean ± SD	51.22 ± 23.12	59.67 ± 27.66	− 8.44 ± 6.96	− 16.50% ± 13.52%	*P* < 0.001
	Range	15.68 to 99.74	15 to 123	− 23.60 to 4.35	− 42.22% to 22.48%	
Corneal pachymetry reduction (μm)	Mean ± SD	51.22 ± 23.12	54.10 ± 27.55	− 2.88 ± 6.74	− 2.33% ± 16.24%	*P* = 0.01
	Range	15.68 to 99.74	10 to 121	− 21.60 to 9.35	− 34.73% to 48.32%	

*SD* = standard deviation

The mean pre- and postoperative central epithelial thicknesses were 53.74 ± 2.54 μm and 59.31 ± 3.38 μm, respectively, signifying a mean thickness increase of 5.56 μm (*P* < 0.001) or 10.50%.

Figure 2b shows the mean pre- and postoperative central epithelial thicknesses.

**Fig. 4:**
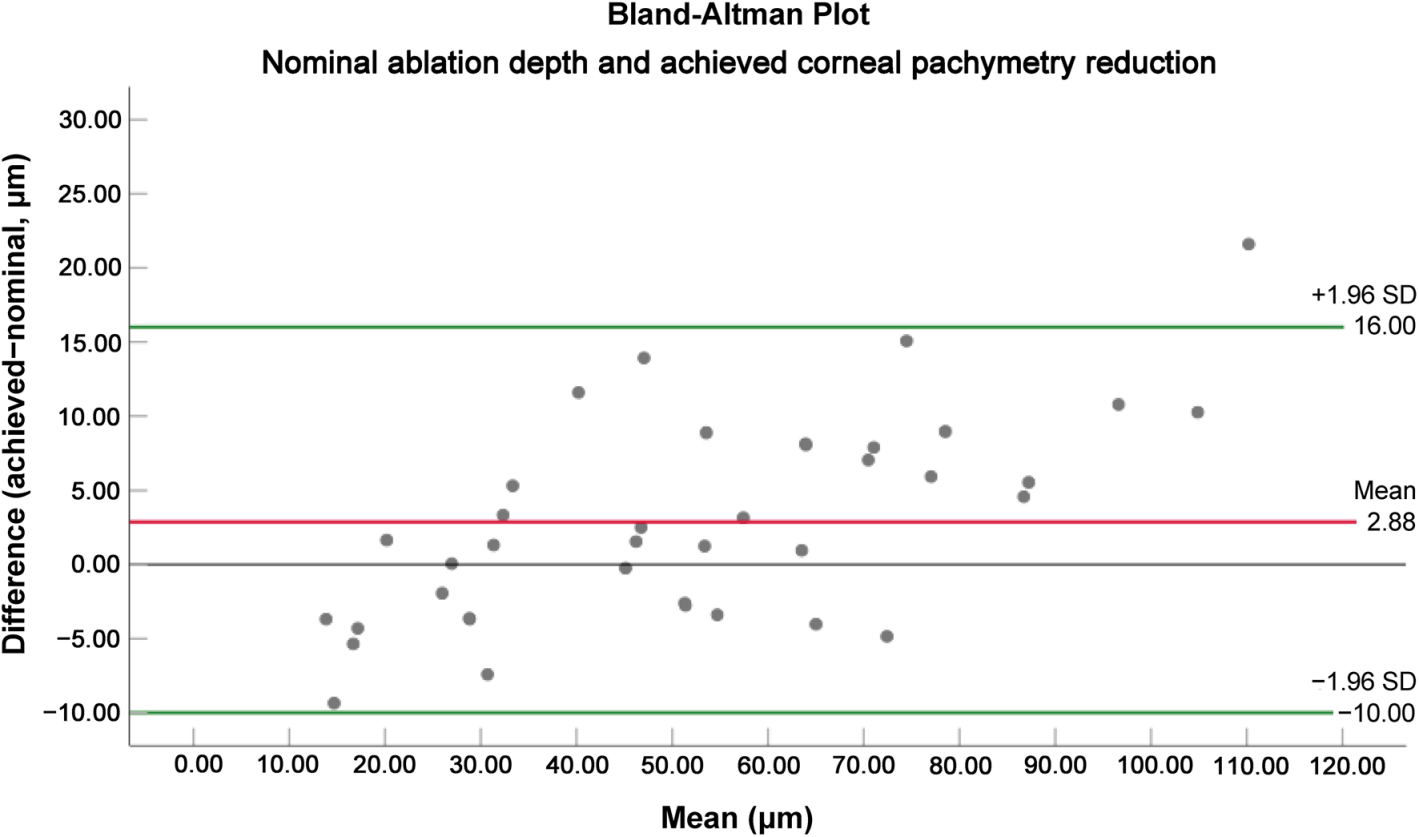
Bland–Altman plot for nominal ablation depth and achieved central corneal pachymetry reduction. The red lines show the mean differences while the green lines show the lower and upper 95% limits of agreement

Because of this increase in the epithelial thickness, the mean postoperative pachymetry reduction (59.67 μm – 5.56 μm = 54.11 μm) was only 2.88 μm or 2.33% greater than the nominal ablation depth (51.22 μm). Pre- and postoperative central corneal thicknesses data are presented in Table 4. Figure 2c shows the mean nominal ablation depth and achieved corneal pachymetry reduction. Figure 4 shows the Bland–Altman plot for nominal ablation depth and achieved corneal pachymetry reduction.

**Table 4:** Pre- and postoperative central corneal thicknesses

Variables		Preoperative	Postoperative	Tissue gain/loss (+ / −)	*P* value
Central pachymetry (μm)	Mean ± SD	520.10 ± 33.82	466.00 ± 37.30	− 54.10 ± 27.55	*P* < 0.001
	Range	459 to 578	390 to 523	− 121 to − 10	
Central stromal thickness (μm)	Mean ± SD	466.35 ± 34.66	406.69 ± 37.83	− 59.67 ± 27.66	*P* < 0.001
	Range	404 to 523	332 to 470	− 123 to − 15	
Central epithelial thickness (μm)	Mean ± SD	53.74 ± 2.54	59.31 ± 3.38	5.56 ± 3.10	*P* < 0.001
	Range	49 to 63	52 to 69	0 to 12	

*SD* = standard deviation

A strong negative correlation was observed between the nominal ablation depth and treated spherical equivalent refraction (SER) (r = – 0.962, *P* < 0.001). A moderate negative correlation was observed between the excessive stromal ablation depth and treated SER (r = – 0.560, *P* < 0.001). No significant correlation was found between postoperative epithelial thickness increase and (A) nominal stromal ablation depth (r = 0.022, *P* = 0.893), (B) treated SER (r = – 0.055, *P* = 0.738), or (C) achieved stromal ablation depth (r = 0.092, *P* = 0.577).

## Discussion

We found a mean central stromal ablation depth of 16.50% greater than the nominal ablation depth, as measured by OCT 6 to 9 months after myopic tPRK, rendering the use of the nominal ablation depth unsuitable for the calculation of RST.

The nominal ablation depth in myopic treatment (decided by the laser ablation software) has normally been assumed to be equal to the depth of the ablated stroma and has been routinely used in calculating the RST. One study from 2020 used the closeness between the nominal ablation depth and the postoperative corneal pachymetry reduction as a confirmation of the validity of the nominal ablation depth for use in RST calculation [3]. Yet, the prevailing understanding of the postoperative increase in central epithelial thickness (resulting from epithelial remodeling after myopic treatment) [9–11] should have prompted the conclusion that a corneal pachymetry reduction close to the nominal ablation depth indicates a greater achieved stromal ablation depth than nominal.

The results from our study in tPRK is the first to objectively demonstrate an excess in central stromal ablation depth. Our results revealed that the traditional calculation of the RST based on the nominal ablation depth appears to be unreliable. This, in turn, compromises the safety of the laser procedures in corneas, where the calculated RST is close to the allowable minimum. As tPRK (and surface ablations generally) are often used for treating cases where LASIK is contraindicated due to thin corneas, the results of the current study, done on tPRK eyes, may be clinically important.

Alcon's StreamLight represents one of the most recent tPRK procedures and has not been extensively reviewed yet [7, 12–16]. As the majority of Alcon laser users presumably do LASIK as their default procedure, they may have only occasional experience with PRK or tPRK. For that reason, a very comprehensive treatment protocol has been outlined in the methods section of the current study.

Several studies have investigated the relationship between the intended (nominal) and achieved ablation depths after LASIK by comparing the nominal central ablation depth with the subsequent reduction in corneal pachymetry. Savini and colleagues [6] examined the agreement between predicted (nominal) ablation depth and pachymetry reduction following femtosecond laser-assisted LASIK for myopia in 85 eyes of 85 patients. They found no statistically significant difference between the mean nominal central ablation depth (66.33 ± 24.15 µm) and the pachymetry reduction at the thinnest corneal location (67.04 ± 30.94 µm), the corneal apex (67.52 ± 31.22 µm), or the pupil center (67.73 ± 31.48 µm). Similarly, Febbraro and colleagues [3] demonstrated that in myopic femtosecond laser-assisted cataract surgery (FS-LASIK), the laser platform's estimation of the maximum ablation (nominal) depth correlated well with the measurement of maximum pachymetry reduction using Scheimpflug. They concluded that it was safe to use the nominal values to calculate the RST.

Corneal epithelial remodeling following myopic laser ablation, initially characterized in 2012 by Reinstein [10] as a compensatory central thickening, has been extensively explored in subsequent studies [11, 13, 17–20]. These investigations have revealed variations in the degree of remodeling associated with different LVC procedures, laser ablation diameters, and ablation profiles [13]. In a report by Shetty and colleagues [13], an analysis of epithelial remodeling differences between tPRK procedures, specifically SmartSurfACE PRK (Schwind eye-tech-solutions GmbH, Kleinostheim, Germany), and StreamLight, indicated a central epithelial thickness increase post-surgery, averaging around 16% at six months for both procedures. de Ortueta and colleagues [21] investigated the refractive impact of epithelial remodeling in myopia after tPRK with the Schwind laser and identified an average excessive corneal pachymetry reduction (compared with nominal ablation depth) of 6 ± 11 µm and a modest increase in central epithelial thickness by 2 ± 4 µm, without mentioning the changes in stromal thickness.

We may speculate that, historically (late 1980s), initial myopia corrections were prone to significant regressions due to the postoperative central epithelial thickening, which is known to be especially pronounced with small-diameter myopic ablations [22]. However, epithelial remodeling was unknown and not measurable at that time. To counteract the regression, we presume that extra laser energy is added to obtain the desired refractive correction; hence, we think an excessive ablation of stromal tissue is programmed to compensate for the opposing effect of the epithelial remodeling and to achieve a corneal pachymetry reduction close to the nominal ablation depth as well as a satisfactory refractive outcome.

Our current study showed that the increase in postoperative central epithelial thickness almost compensated for the excessive ablated in stromal tissue, yielding a reduction of total central pachymetry quite close to the nominal ablation depth. This resulted in a predictable alteration in the corneal anterior shape and provided favorable refractive results.

Chen and colleagues' study [22] reported a significant thickening of the epithelium with a high amount of programmed spherical equivalent correction. The absence of this correlation in our study may be attributed to our small sample size and the relatively modest treated spherical equivalent. Our findings revealed a significant negative correlation between corrected refraction and excessive stromal ablation depth, indicating that higher levels of myopia lead to increased excessive stromal tissue removal. This emphasizes the need for cautious approach in high myopic corrections to ensure safe RST, minimizing the risk of complications such as iatrogenic ectasia.

While our study elucidates the discrepancy between nominal and achieved stromal ablation depths, it is crucial to acknowledge the possibility of stromal remodeling and tissue deposition post-PRK, which may contribute to further alterations in corneal thickness. Previous studies have documented stromal remodeling following PRK. Although one study [23] reported a decrease in keratocyte density in the anterior stroma for at least five years post-PRK, another study [24] found that stromal thickness remained unchanged between 1 and 36 months post-PRK.

Previous studies have highlighted factors like epithelial irregularity, postoperative haze, and interface reflectivity changes affecting the reliability of SD-OCT [25]. Although post-PRK corneas may introduce some measurement variability, in the absence of haze, our study should not be affected by the mentioned factors. Furthermore, our earlier published paper reported good repeatability of Avanti SD-OCT in measuring virgin, post-LVC and keratoconic eyes [26]. Therefore, our study's insights into the discrepancy between nominal and achieved stromal ablation depths post-PRK remain valid.

Our finding of the excess ablation of the stroma challenges the viability of the preoperative calculation of RST based on the nominal ablation depth. This implies that adhering to older RST standards, such as a minimum of 250 µm, may increase the risk of iatrogenic keratectasia, a formidable complication in laser refractive surgery [27].

There are some limitations to be taken into consideration. The sample size of 40 eyes may limit generalizability, and the retrospective component introduces potential selection bias. Additionally, Avanti SD-OCT, may have limitations in accurately mapping corneal thickness post-PRK. The central pseudo-Bowman’s membrane post-PRK is both thinner and more variable in thickness compared with a virgin Bowman’s membrane [28], which may have affected the quality of the stromal measurements in our study. Conducting the study at a single center further limits generalizability, and the follow-up duration of 6 to 9 months may not capture long-term changes. Addressing these limitations through larger sample sizes, prospective designs, different imaging technologies, multicenter collaborations, and longer follow-up durations could enhance the quality of the future research in this area.

## Conclusions

In low and moderate myopic StreamLight tPRK, the mean central stromal removal, as measured by OCT, was found to be 8.44 µm greater than the nominal ablation depth specified by the laser ablation planning software, signifying a stromal ablation excess of 16.50%. On the other hand, due to the increase in epithelial thickness postoperatively, we measured only a small (2.33%) mean difference between the central nominal ablation depth and the postoperative pachymetry reduction. This small difference has also been reported in other LVC procedures and is an indication of predictable refractive outcomes; however, it cannot be interpreted as evidence for the safe use of nominal ablation depth in calculating RST.

Hence, we advise that the nominal central ablation depth in myopic treatments should only be looked upon as an indicator for postoperative central corneal pachymetry reduction and not as a measure of real central stromal ablation depth. Future studies assessing the reduction of stromal thickness after current myopic LVC techniques are needed, and the laser producers are urged to provide more accurate stromal ablation depth value for RST calculations. To know the real RST, especially in correcting high myopia or astigmatism in eyes with low central pachymetry, it should be imperative to prevent unwanted iatrogenic ectasia.

## Data Availability

The data that support the findings of this study are available from the corresponding author upon reasonable request.
